# Congeners-Specific Intestinal Absorption Of Microcystins In An *In Vitro* 3D Human Intestinal Epithelium: The Role Of Influx/Efflux Transporters

**DOI:** 10.3389/ftox.2022.883063

**Published:** 2022-08-05

**Authors:** Laura Turco, Nicoletta Santori, Franca M. Buratti, Jean-Lou C. M. Dorne, Emanuela Testai

**Affiliations:** ^1^ Istituto Superiore Di Sanità, Environment & Health Dept, Rome, Italy; ^2^ EFSA (European Food Safety Authority), Parma, Italy

**Keywords:** cyanotoxins, microcystin congeners, transporters, intestinal absorption, influx/efflux proteins

## Abstract

Microcystins constitute a group of over 200 variants and are increasingly considered as emerging toxins in food and feed safety, particularly with regards to sea-food and fish consumption. Toxicity of MCs is congener-specific, being characterised by different acute potencies, likely related to the differential activity of metabolic enzymes and transporters proteins involved in their cellular uptake. However, the active transport of MCs across intestinal membranes has not been fully elucidated. Our results, obtained using a fit for purpose 3D human reconstructed intestinal epithelium, provide new information on the complex mechanisms involved in the absorption of 5 MC variants’: it is indeed characterised by the equilibrium between uptake and extrusion, since the selected congeners are substrates of both influx and efflux proteins. In the range of tested nominal concentrations (10–40 µM) fully representative of relevant exposure scenarios, none of the active tested transporters were saturated. The comparison of permeability (Papp) values of MCs variants highlighted a dose independent relationship for MC-LR, -YR and -RR (Papp x 10^–7^ ranged from 2.95 to 3.54 cm/s), whereas -LW and–LF showed a dose dependent increase in permeability reaching Papp values which were similar to the other congeners at 40 µM. MC-RR, -LR, -YR show absorption values around 5% of the administered dose. Due to their lipophilicity, MC-LW and -LF were also detected within the cellular compartment. The intestinal uptake was only partially attributable to OATPs, suggesting the involvement of additional transporters. Regarding the efflux proteins, MCs are not P-gp substrates whereas MRP2 and to a lesser extent Breast cancer resistance protein are active in their extrusion. Despite the presence of GST proteins, as an indication of metabolic competence, in the intestinal tissue, MC-conjugates were never detected in our experimental settings.

## 1 Introduction

Over the last decades, anthropogenic activities and climate changes have increased the extension and frequency of cyanobacteria growth. Indeed, cyanotoxins have been referred to as emerging risks for human health (Buratti et al., 2017; Chorus and Welker, 2021) particularly in the food and feed safety areas (Testai et al., 2016). Microcystins (MCs) are a group of cyanobacterial toxins frequently detected in freshwaters, consisting of more than 200 variants, each characterised by different amino acid combinations and other structural changes (e.g. methylation/desmethylation of several functional groups) (Spoof and Catherine, 2017). The toxicity of MCs is congener-specific being characterised by a range of acute toxic potencies, likely related to the toxicokinetic properties of the single congeners (Santori et al., 2020). Indeed, the detoxification pathway, via enzymatic or spontaneous glutathione (GSH) conjugation (Buratti and Testai, 2015; Santori et al., 2020), as well as the active transport, responsible for the uptake into cells, appears to be congener-dependent (Fischer et al., 2005, 2010). Conversely, the *in vitro* inhibition of protein serine/threonine phosphatases (PPPs), the key molecular event of MCs’ Mode of Action, is comparable amongst the congeners (Hoeger et al., 2007; Monks et al., 2007; Fischer et al., 2010; Vesterkvist et al., 2012), with differences in sensitivity among the various PPPs (Altaner et al., 2019).

MCs have been shown to be human organic anionic-transporting polypeptides OATPs substrates in several *in vitro* studies (Fischer et al., 2005, 2010; Niedermeyer et al., 2014; Bulc Rozman et al., 2017; Kaur et al., 2019). The OATP family of transporters consists of 11 members with OATP1A2, OATP1B1, OATP1B3, and OATP2B1 being the most extensively characterised and involved in the disposition of drugs and xenobiotics (König et al., 2006; Zhang and Lauschke, 2019). The various OATPs are differentially expressed in various tissues such as enterocytes, hepatocytes, kidney cells and the blood–brain barrier (Feurstein et al., 2009), thus explaining the multi-organ toxicity of MCs particularly since their bioavailability in target cells is dependent on the activity of influx/efflux transporter proteins. In the liver, the toxicity of MC variants is potentially affected by the differential uptakes mediated largely by OATP1B1 and 1B3 (Fischer et al., 2005, 2010). Scant data have been generated with regard to efflux proteins involved in MC extrusion (Kaur et al., 2019) and the picture is even more complex since cellular export of MC-conjugates has not been depicted to date.

Hence, the passage of MCs through the intestinal barrier represents the first kinetic step after human oral exposure. Among OATP family members, 1A2 and 2B1 are the most represented in the luminal side of the enterocytes (Kalliokoski and Niemi, 2009; Shitara et al., 2013) although Gameiro et al. (2017) reported that OATP2B1 is also present in the basolateral side in developmental stages ranging from the infant to the teenager. P-glycoprotein (P-gp), Multidrug resistence protein 2 (MRP2) and Breast cancer resistance protein (BCRP) have been reported to be the major efflux transporters expressed in the small intestine (Takano et al., 2006; Misaka et al., 2013).

*In vitro* studies on transport kinetics are usually based on experimental models that are not fully representative of the actual and complete framework of transporter systems present in the human intestinal epithelium. Indeed, for cellular drug uptake studies, *in vitro* transfected systems (cells or vesicles) overexpressing a single specific transport protein are usually used (Kaur et al., 2019). Such studies aim to demonstrate if a specific chemical is a substrate of the expressed transporter, providing an unequivocal rationale for the interpretation of results, with the limitation of potential loss of the possible competition with other transporters. Considering more complex systems, kinetic studies of *in vitro* intestinal absorption are usually performed with intestinal cells grown on trans-well microporous filters.

Available data on the MCs intestinal absorption have been mostly carried out using Caco-2 cell line and are characterised by a high variability, probably due to the variable patterns of OATP expressions in this cell line (Zeller et al., 2011; Henri et al., 2014). Inter- and intra-laboratory variability are widely reported as the main limitation for such as a cell line in active transport kinetic studies due to the dependence of transporter expressions on culture conditions and the number of passages for the culture (Oltra-Noguera et al., 2015).

The aim of this study is to describe the intestinal absorption kinetics of some MC congeners (MC-LR, -RR, -YR, -LW and -LF) as well as their GSH conjugates, using an *in vitro* model which is as close as possible to the human intestinal barrier, i.e. a 3D system of intestinal epithelium (EpiIntestinal™). This commercially available *in vitro* model has been reconstructed from normal human primary intestinal cells (epithelial cells, fibroblasts and endothelial cells) grown onto microporous membrane inserts and closely mimics the physiology, 3D tissue architecture, and function of the small intestine (Ayehunie et al., 2018). After assessing the expression and activity of the relevant intestinal transport proteins, this *in vitro* experimental model allowed us to study the complex kinetics of MC variants in the intestine investigating the involvement of influx (OATPs) and efflux transporters (such as P-gp, MRP2 and BCRP) and Glutathione-S-transferases-mediated metabolism at the same time.

## 2 Materials and Method

### 2.1 Chemicals

Microcystin-RR (MC-RR), Microcystin-LR (MC-LR), Microcystin-YR (MC-YR), Microcystin-LF (MC-LF) and Microcystin-LW (MC-LW), isolated from Microcystis auriginosa purity ≥95% were obtain from Enzo Life Sciences (Farmingale, NY); Digoxin, Paracetamol, Narigin and Verapamil were purchased from Sigma Aldrich with purity >97.4%, >99%, >95% and >98% respectively. Also Benzbromarone (purity≥92,5%), Cyclosporin (purity >97%), Atorvastatin (purity >98%), Pravastatin (purity >98%) was bought Sigma-Aldrich. Methanol, Lucifer Yellow and trifluoroacetic acid were purchased from commercial source.

### 2.2 *In Vitro* Intestinal System

EpiIntestinal™ microtissues grown on transwell inserts were obtained from MatTek (Bratislav, Slovakia) and maintained according to the manufacturer’s protocols. The used kits consist of 24 individual tissues cultured in cell culture inserts; dimensions: 9 mm tissue diameter (surface area = 0.6 cm^2^), underlying microporous membrane pore size = 0.4 μm; insert material: polycarbonate. Structural analysis of EpiIntestinal confirms a polarized epithelium with villi structures, with the apical (AP) and the basolateral (BL) compartments mimicking the intestinal lumen and the blood circulation, respectively. For transport kinetics, fresh tissues were used the day after their arrival following the typical experimental scheme for transport studies with epithelia grown on transwell inserts. The substrate compound is administered in the donor compartment (AP or BL, depending on the experiment), than the amount of the compound is detected at the end of the incubation in the receiver compartment at the opposite side. Reconstructed tissues from the same donor were used in order to characterise the role of transporters in MCs’ transport, excluding the inter-individual variability as a possible confounding factor. Independent experiments were carried out with different tissue lots (the suppliers produces a new lot each week; kits within a production lot are identical in regards to cells, medium, handling, culture conditions).

### 2.3 Protein Identification by Western Blot Analysis

We used a gel gradient with stain-free gel technology, a system enabling visualization of proteins band in gels and transfer membranes without using colorimetric or fluorescent stains or requesting the use of housekeeping proteins.

Cells were solubilized by RIPA buffer and then proteins were fractionated by SDS-PAGE with 8–16% polyacrylamide gradient gel. All the lines were charged with the equal amount of the same sample; two lanes were devoted to charge the molecular weight control proteins. Proteins were transferred onto a nitrocellulose membrane by electroblotting. Then the membrane was cut into separated parts to allow incubation with different antibodies. The membranes were maintained in blocking solution (Blocker solution BSA in TBS from Thermo Scientific) for 4 h, washed and then incubated overnight at 4 °C with primary antibodies at several dilutions (as recommended by suppliers for Western Blot analysis). Monoclonal antibodies were used to detect human BCRP (Santa Cruz), MRP and GST (Sigma) while polyclonal antibodies were used against P-gp and OATP 1A2 and 2B1 (Sigma).

Membranes were washed and incubated with horseradish peroxidase-conjugated IgG (rabbit, anti-mouse or mouse anti-rabbit, depending on primary antibody) for 1 h and were then stained using the ECL detection kit reagents (Pierce ECL plus from Thermo Scientific). Protein concentration was determined using the Bradford method (Bradford, 1976).

### 2.4 *In Vitro* Transport Kinetics

MCs, reference substrates and inhibitors were dissolved in DMSO and were then diluted in transport buffer (10 mM glucose in 10 mM HEPES buffer); to mimic the actual *in vivo* conditions of both compartments two pH gradients (pH 6.5 in AP and pH 7.4 in BL) were applied. The final concentration of DMSO was kept at 1% to avoid solvent toxicity. AP and BL volumes were 200 and 500 μL, respectively. Tissues were incubated with the compounds for up to 4 h (time points: 0, 30, 60, 120 and 240 min) at 37°C to check the linearity in dependence on the incubation time. Aliquots (100 µL) of the AP medium were analysed, to derive the test item concentration at T0 and then, for the other time points, aliquots (50 µL) of BL medium (replacing with the same amount of fresh medium) were analysed to measure toxin concentration in the receiver compartment. MC nominal concentrations were 10, 20 and 40 μM; for the main experiments using inhibitors, considering the distribution among the three compartments, 40 μM at 4 h of incubation were selected in order to have adequate analytical sensitivity.

To assess the barrier integrity, as a measure of treatment-related toxicity, the impermeable dye Lucifer Yellow (100 µM) was applied at the AP side of each tissue. Lucifer Yellow (LY) is a fluorescent hydrophilic small molecule that does not interact with cell components (Stewart, 1981); its passive paracellular transport across biological barrier reveals damages to functional tight junctions and for this reason it is widely used in transport studies (Silve et al., 2012). At the end of any incubation, aliquots (0.3 ml) were taken from the receiver compartment and analysed: any fluorescence due to LY presence was used as an excluding criterion for the tissue. After 4 h, samples from all the compartments of the system (AP, BL and cellular compartment) were harvested for HPLC detection. The scraped cells were dissolved with 100 µL MeOH and vortexed for 30 s; cell lysate was then centrifuged (Eppendorf 5417R; 5 min, 4°C, 16000 g) and the upper phase was analysed by HPLC system.

Mass balance (MB), expressed as the % of the test item recovered at the end of the experiment, was defined as the sum of the amount of each test item recovered in the AP and BL compartment, as well as the fraction remaining associated to the cellular fraction. A MB ≤ 70% was set as not acceptable, in order to have a reliable value for absorption. At least two independent experiments on different tissue lots (biological replicates) from the same donor have been conducted, each of them in duplicates (technical replicates).

Specific inhibitors were used in the standard incubation by adding them 15 min before (as pre-incubation) and during the 4 h kinetics together with the substrates.

The following pair of compounds were used as reference substrate/inhibitor of P-gp, MRP, OATP 1A2 and OATP 2B1 respectively: Digoxin (100 µM)/Verapamil (50 µM), Atorvastatin (100 µM)/Benzbromarone (100 µM), Fexofenadine (10 µM)/Naringin (50 µM) and Atorvastatin (100 µM)/Cyclosporin (10 µM). Testosterone (100 µM) was used in bidirectional (apical to basal compartment and vice versa) transport experiments to highlight BCRP activity being testosterone a substrate of BCRP only. The concentrations of inhibitors to be used were derived from literature IC_50_/Ki data or *in vitro* studies with the same chemical (Kalliokoski and Niemi, 2009; Jouan et al., 2016; Nicolaï et al., 2017).

### 2.5 High Performance Liquid Chromatography (HPLC) Method

All substrates used were analysed and quantified by ad hoc HPLC-UV methods, using C-18 DB reversed-phase column (length = 25 cm, diameter = 4.6 mm). Conditions for analysis were set up and were as follows: 1 ml/min flow rate, 20 μL injection volume.

MCs and the other compounds were identified and quantified using the method described in Buratti et al. (2011) and Buratti et al. (2013) with minor modifications. Briefly mobile phase consisting of MeOH/H_2_O mixture (both containing 0.05% trifluoroacetic acid (TFA)): 70:30 (v/v) and *λ* = 238 nm was used for detection of MC-LR, MC-YR, MC-LW, MC-RR, MC-LF, with the respective GSH conjugates, Atorvastatin and Pravastin; 60:40 (v/v) and *λ* = 220 nm was used for Digoxin and 10:90 and *λ* = 254 nm for Paracetamol.

The quantification of all substances was carried out using commercial standard to build a calibration straight line prepared with 5 or 7 concentrations tested within the range 0.5–100 μM (MC-LR: *R* ^2^ = 0.998, MC-YR: R2 = 0.9825, MC-LW: *R* ^2^ = 0.9934, MC-RR: *R* ^2^ = 0.9952, MC-LF: *R* ^2^ = 0.9961, Atorvastatin: *R* ^2^ = 0.9412; Pravastin: *R* ^2^ = 0.9950, Digoxin: *R* ^2^ = 0.9956, Paracetamol: *R* ^2^ = 0.9981). Since a commercial standard for the GSH-MCs conjugates is not available, they were chemically obtained in extreme alkaline condition to build up the respective calibration curves (Buratti et al., 2011, 2013; Santori et al., 2020). The coefficient of variation of the methods was <10%.

### 2.6 Data Analysis

Kinetic results for absorption are expressed as Apparent Permeability coefficients (Papp) both in apical to basal direction (Papp, AP-BL) and in basal to apical direction (Papp, BL-AP) and derived as follows:

$$\text{Papp}=\frac{Q}{t\;S\;C0}\;\;$$

where Q is the amount of compound recovered in the receiver compartment after the incubation at time t, C0 the initial compound concentration given to the donor compartment, and s the surface area of the Trans-well inserts (Santa-Maria et al., 2020). The actual measured concentrations at T0 were used for the calculation rather than for the nominal concentrations. This approach was relevant since throughout the experiments deviations from the nominal concentrations were in the range of ±32%. Efflux ratio (ER) is calculated as the quotient of Papp BL-AP to Papp AP-BL.

Data are presented as the mean ± standard deviation. The difference between the groups was analyzed using the paired Student’s t-test. *p* < 0.05 was considered to indicate a statistically significant difference. GraphPad 6 Prism™ software was used to obtain the linear regression and the statistical calculations.

## 3 Results

### 3.1 Absorption Kinetics of Five Microcystins

As first step, 5 MC-congeners (LR, LW, YR, RR and LF) were tested in order to rank their ability to cross EpiIntestinal™ tissues. Preliminary results highlighted that in the range of MC nominal concentrations (10, 20, 40 µM), no toxicity, measured as barrier integrity with LY, was observed. Indeed, after 4 h kinetic testing, no fluorescence has been detected in the receiver compartments in any of the experiments. The absolute amount recovered in the BL compartment at 4 h was linearly dependent on MC concentrations for MC-RR, –YR and–LR, with a constant percentage of 3.6, 4.3 and 4.6% of the applied dose, respectively. The passage into the BL compartment was not detectable at 10 µM for MC-LF and–LW and the % absorbed increased with increasing toxin concentrations, indicating that a steady state was not reached and transporter are not saturated.

All MB values were within the acceptability criteria (≥70%), with the great majority of MCs detected in the AP compartment. Interestingly, for the more hydrophilic variants recovery was around 85–90%, while the lowest values (around 70–80%) were obtained with the most lipophilic ones (-LF and -LW), Only these two latter variants were recovered in the cellular pellet, although in a very small amount (less than 1% of the administered dose).

**FIGURE 1:**
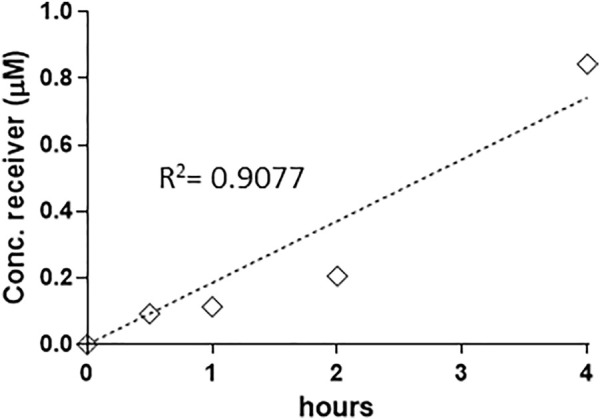
Linear increase of MC-RR (40 µM) absorption up to 4 h (0.5, 1, 2 and 4 h). Absorption has been defined as the recovery (expressed as concentration in the receiver BL compartment), since no toxin was detected in the cellular compartment. The permeability rate value (Papp AP-BL) is Papp x 10^–7^ 5.73 cm/s. Two independent experiments on different tissue lots from the same donor have been carried out each in duplicates.

When a time course was conducted (MC conc. = 40 µM), MC-RR appeared to be the most rapidly uptaken and absorbed, since it was detectable in the receptor fluid already after 30 min: the concentration then linearly increased up to 4 h, with permeability coefficient Papp x 10^–7^ = 5.73 cm/s (Figure 1). For the other MC variants, the absorption was not linear vs. time and a lag phase was evident, with toxins detected in the receptor fluid only after 2 h (data not shown). On this basis, 40 μM and 4 h incubation were selected for the other analysis to maximise the sensitivity of the system.

**FIGURE 2:**
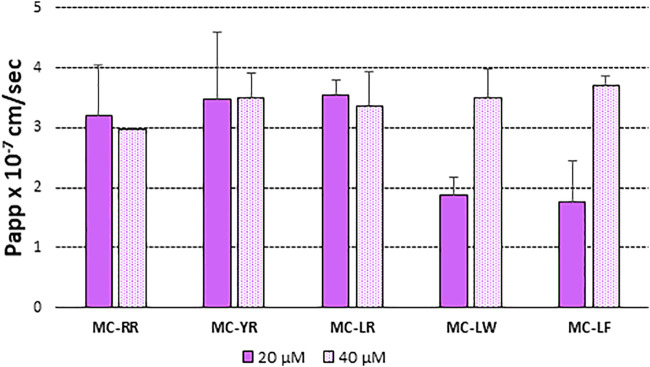
Papp (AP-BL) values of: MC-RR, MC-YR, MC-LR, MC-LW and MC-LF at 20 µM (closed bar) and e 40 µM (open bar). MCs order was based on increasing lipophycility (Santori et al., 2020). At least two independent experiments on different tissue lots from the same donor have been carried out each in duplicates. Results are expressed as means ± standard deviations.

Figure 2 illustrates differences in the Papp values at 20 and 40 µM measured as the amount of each MC variant recovered at the receiver (BL) compartment of the Transwell system after 4 h of incubation. This approach allowed measuring the net resultant of several, even opposite, actions (efflux and influx). Papp values were independent on the donor concentrations for MC-LR, -YR and -RR (Papp x 10^–7^ ranged from 2.95 to 3.54 cm/s), while dose dependent increase in permeability was highlighted for -LW and–LF (the more lipophilic ones) for which the Papp x 10^–7^ values reached values similar to the other congeners only at 40 µM toxin.

### 3.2 Presence and Activity of Transporters in the Test System

Although the provider of the tissues generally indicates the presence of P-gp, MRPs and BCRP transporters in EpiIntestinal™ (Cui et al., 2020), we first characterised our experimental model, to evidence that our donor was not an exception, due to specific individual characteristics. We also included in the analysis the OATPs (1A2 and 2B1) transporters, potentially relevant for MC uptake and GST proteins involved in MCs biotransformation. The Western Blot analysis indicating that EpiIntestinal™ tissues express all the influx and efflux transporters that are considered relevant as well as GST proteins, as the major biotransforming enzyme for MCs (Supplementary Figure S1).

**FIGURE 3:**
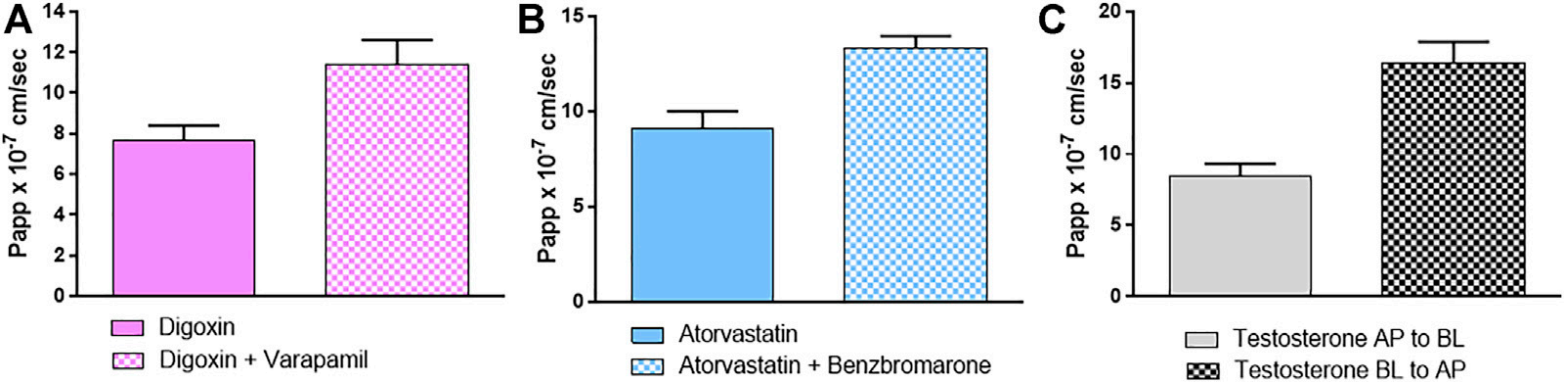
Papp (AP-BL) values of specific probe substrates with and without specific inhibitors **(A)** digoxin/verapamil **(B)** atorvastatin/benzbromarone as substrate/inhibitor of P-gp and MRP respectively and **(C)** Papp (AP-BL) and (BL-AP) of testosterone used as BCRP probe substrate. Details are given in M&M. Results are expressed as means ± standard deviations.

In addition, specific probe substrates (S) and inhibitors (I) were used to depict transporter activity. Concerning efflux transporters, Digoxin (S) and Verapamil (I) were selected for P-gp, while Atorvastatin (S) and Benzbromarone (I) were used for MRP. Results in Figures 3A,B show that both P-gp and MRP were active in extruding their probe substrates and their activity was suppressed when the specific inhibitors were applied, as evidenced by an increase in the amount of substrates recovered at the BL compartment.

The activity of BCRP was indirectly demonstrated by investigating the balance of the bidirectional passage through the epithelium using testosterone as a highly specific substrate for BCRP. As Figure 3C shows, the significant passage of testosterone from BL to AP compartment suggests the activity of the efflux transporter BCRP: indeed the ER is = 1.95, meaning that the efflux system predominates.

**FIGURE 4:**
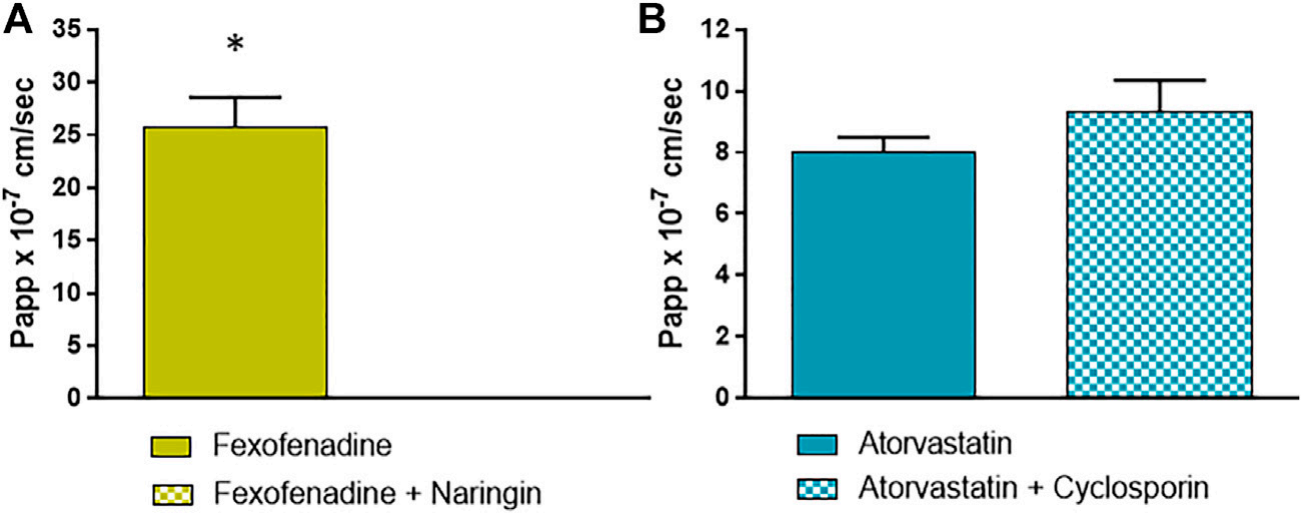
Papp values of **(A)** fexofenadine with and without naringin as probe substrate and inhibitor of OATP 1A2; and **(B)** atorvastatin with and without cyclosporin as probe substrate and inhibitor of OATP 2B1. Details are given in M&M. Results are expressed as means ± standard deviations. Stars represent statistical significance of the results with *p* < 0.05.

For OATP 1A2 and 2B1, substrate/inhibitor systems as fexofenadine/naringin and atorvastatin/cyclosporin were used, respectively (Grube et al., 2006; Ho et al., 2006; Kalliokoski and Niemi, 2009) (Figures 4A,B). Figure 4A shows that OATP 1A2 is active in EpiIntestinal™ tissues, as evidenced by fexofenadine absorption, which is also completely prevented by the inhibitor naringin. The significant recovery of atorvastatin in the receptor fluid clearly indicates that OATP 2B1 is also active (Figure 4B), but the OATP inhibitor cyclosporin use induced an increase in the absorption, rather than its decrease. This could be explained by the poor selectivity of the couple substrate/inhibitor used for this transporter. In fact, cyclosporin inhibits OATP 2B1 and the cyclosporin-sensitive efflux systems, such as P-gp and MRP2; consequently the effects of the OATP inhibition could be overshadowed (Misaka et al., 2013).

### 3.3 Involvement of Transporters in Microcystin Absorption

**FIGURE 5:**
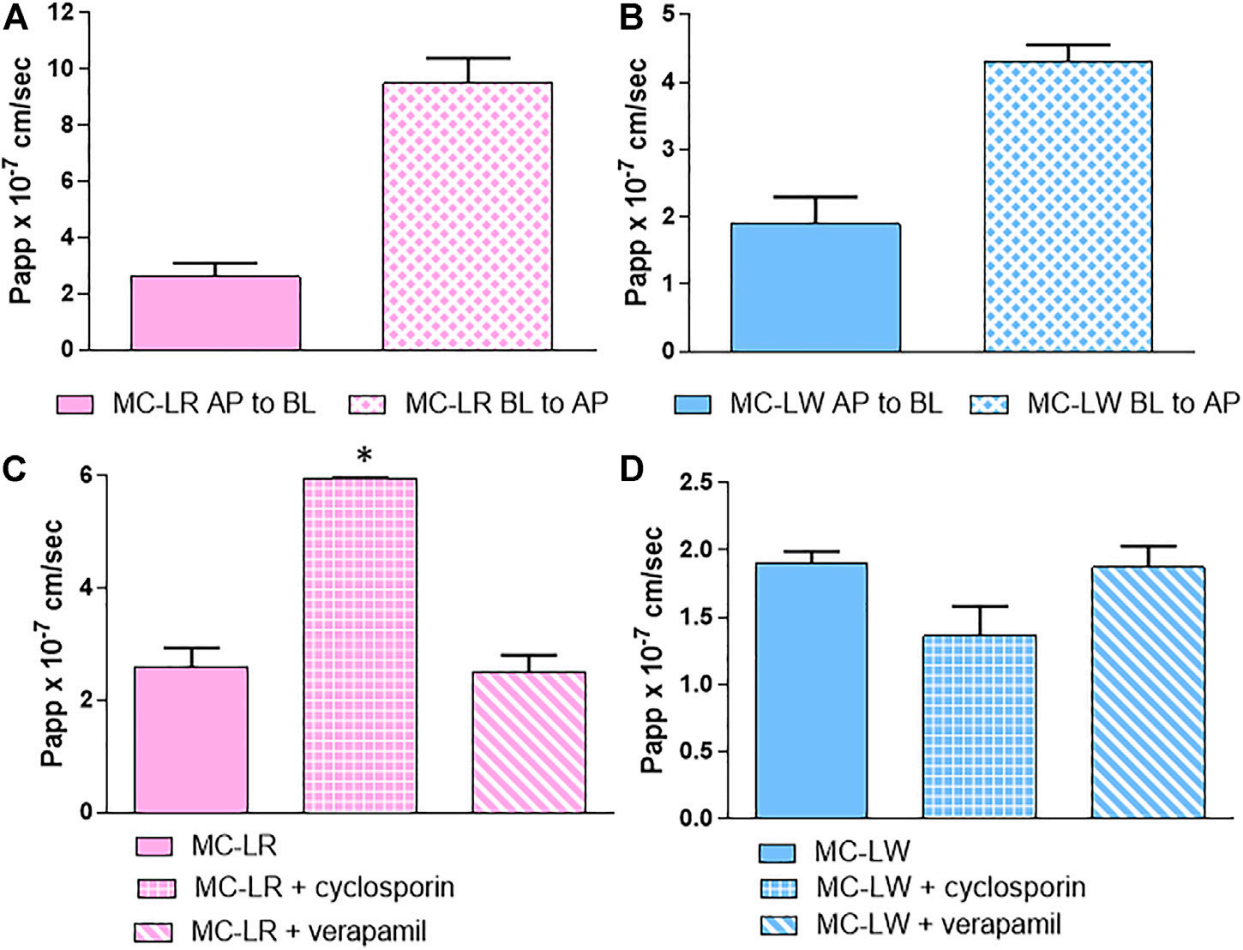
Bidirectional absorption kinetic: Papp values (AP-BL) and (BL-AP) of **(A)** MC-LR and **(B)** MC-LW. Papp values of MC-LR **(C)** and MC-LW **(D)** with and without cyclosporin and verapamil: from left to right MC without inhibitor, MC with cyclosporin and MC with verapamil. Toxin concentration was 40 μM; study duration was 4 h. At least two independent experiments on different tissue lots from the same donor have been carried out, each in duplicates. Results are expressed as means ± standard deviations. Stars represent statistical significance of the results with *p* < 0.05.

In order to check which transport systems were involved in the MCs overall intestinal absorption kinetic, we firstly checked if MCs are substrates of some of the efflux systems present in our model. The bidirectional passage of MC-LR and -LW (Figures 5A,B), taken as representative of hydroliphilic and lipophilic variants, confirms that these congeners are substrates of both influx and efflux proteins. The ER was 3.65 and 2.65 for MC-LR and MC-LW, respectively: since in both cases the ratio was >1, the efflux system predominates over the influx.

The results were not affected by the co-administration of verapamil, a P-gp inhibitor, while cyclosporin significantly decreased the efflux of MC-LR and although not reaching the statistical significance the influx of MC-LW (Figures 5C,D). MC-LR and -LW are therefore likely substrates of MRP2, whose activity is cyclosporin dependent, but not of P-gp.

**FIGURE 6:**
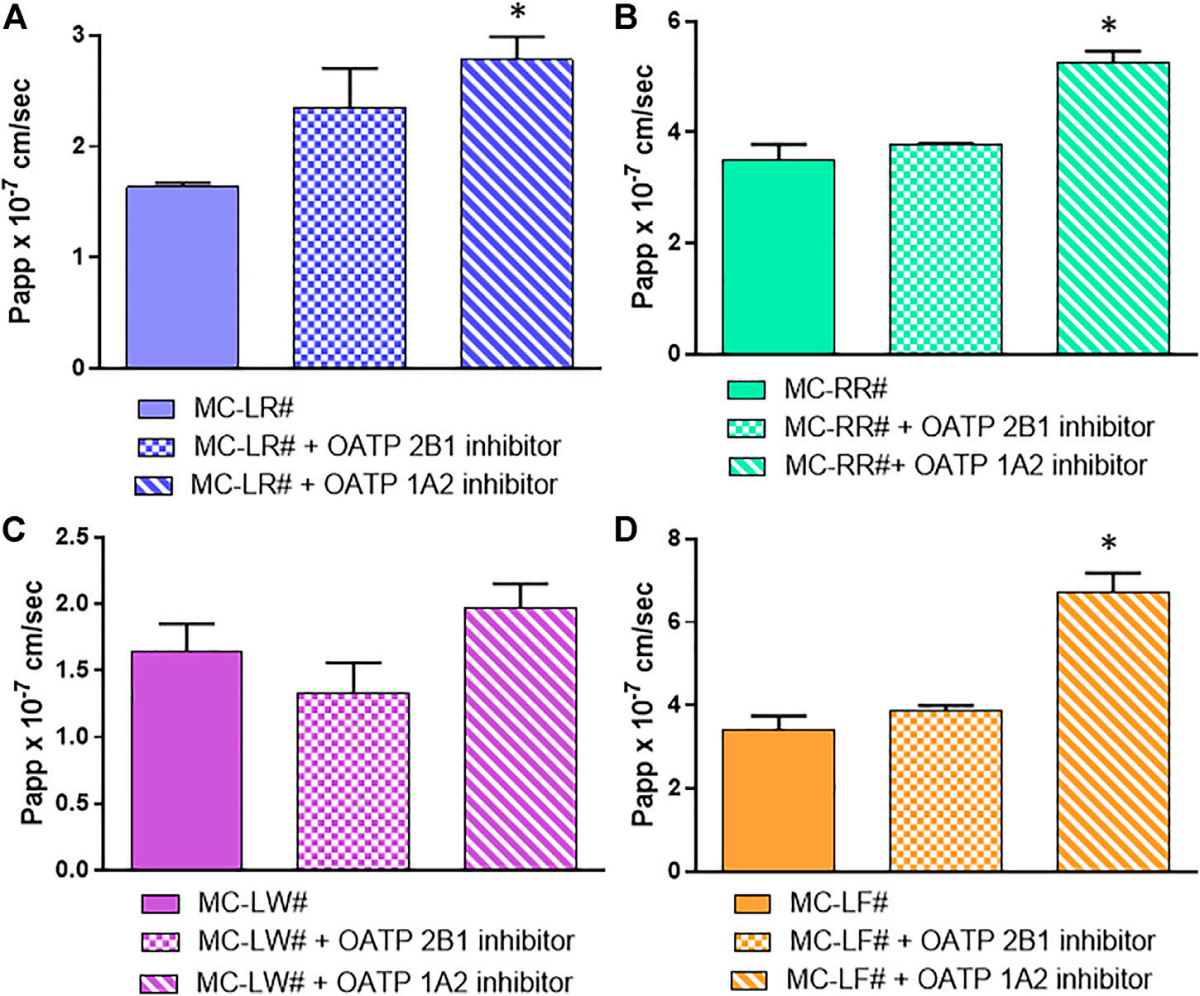
Involvement of influx protein in MCs absorption. **(A)** MC-LR **(B)** MC-RR **(C)** MC-LW and **(D)** MC-LF. Toxin concentration was 40 μM; study duration was 4 h. The 3 bars in each section of the figure represent from left to right: Papp values of MCs in the presence of Digoxin plus Benzbromarone (as P-gp, MRP and BCRP inhibitors) set as the control (#); control mixture with 100 µM Pravastatin as OATP2B1 inhibitor; control mixture with 50 µM Naringin as OATP1A2 inhibitor. At least two independent experiments on different tissue lots from the same donor have been carried out, each in duplicates. Results are expressed as means ± standard deviations. Stars represent statistical significance of the results with *p* < 0.05.

In order to see the role of the OATPs in MC uptake, we used specific inhibitor-cocktails in co-administration with MCs to have a single OATP (1A2 or 2B1) active, time by time, with the selected efflux blocked. The addition of digoxin/benzobromarone inhibits the three efflux systems (Takano et al., 2006; Ursic et al., 2009); while naringin and pravastatin (100 µM) muted OATP 1A2 and 2B1 activity, respectively (Bailey et al., 2007; Shitara et al., 2013). Pravastatin has been used as OATP 2B1 inhibitor due to the inhibitory action of cyclosporin on both influx and efflux transporters. Results in Figure 6 show that the use of pravastatin almost unaffected the uptake: only MC-LW influx was slightly decreased when pravastatin was used as OATP 2B1 inhibitor (Figure 6C), although the difference was not statistically significant. Conversely, when naringin was used as OATP 1A2 inhibitor, a significant increased amount of MCs was recovered in the BL compartment (Figure 6) which can be explained by an efflux activity inhibition.

### 3.4 Biotransformation of Microcystins in EpiIntestinal™

Despite the presence of GSTs, in none of the experiments, MC-glutathione conjugates were detected in the AP or BL compartment, nor in the cellular pellet harvested at the end of the experiment.

## 4 Discussion

Although data on MCs’ occurrence in food items are still limited, due to the use of inadequate analytical tools (Testai et al., 2016; Chorus and Welker, 2021), the available information suggests that MCs represent an emerging risk in the food and feed safety areas, particularly when sea food and fish are concerned (Testai et al., 2016). Therefore, knowledge of their intestinal absorption to estimate oral bioavailability is a critical step for their risk assessment. MCs show congener-specific acute toxicity, which have been related to differential affinity towards xenobiotic-metabolising enzymes and transporter proteins involved in their cellular uptake (Altaner et al., 2019; Santori et al., 2020; WHO, 2020).

A passive transport has been mainly excluded for MCs (WHO, 2020). Most of the studies investigating MCs’ active transport have been carried out in hepatocytes to elucidate their cellular uptake in the liver, considered as the major target for MC toxicity. Indeed, the expression of OATP1B1 and OATP1B3 on the basolateral (sinusoidal) side of hepatocytes is responsible for MCs’ uptake by the liver (Fischer et al., 2005; 2010) in a congener-specific pattern: MC-LW and MC-LF uptake is higher than MC-LR, and even lower uptake was measured for MC-RR (Fischer et al., 2010). OATP1A2 has been identified as the responsible MC transporter at the blood–brain barrier (Fischer et al., 2005). These findings pertain mainly the distribution phase: MC active transport across intestinal membranes have not been fully elucidated. Available data suggest that OATPs may be involved, but the specific OATP isoform responsible for intestinal absorption of MCs have yet to be identified (WHO, 2020).

For this reason, we carried out the present study with the aim of providing new information on MC absorption by using a 3D experimental model as a reconstructed human intestinal epithelium (EpiIntestinal™), from normal cells, as a relevant proxy mimicking the physiology, tissue structure, and function of the epithelium of the small intestine (Ayehunie et al., 2018).

By Western Blot analysis and kinetic evaluation with specific substrate/inhibitor pairs, the expression of active influx and efflux proteins, most represented in the human intestinal epithelium (OATP 1A2 and 2B1 as influx proteins and P-gp, MRP2 and BCRP as efflux transporters), was confirmed in the EpiIntestinal™ tissues used in this study (Takano et al., 2006; Kalliokoski and Niemi, 2009; Shitara et al., 2013). This evidence demonstrates the relevance of this *in vitro* model, endowed with the overall panel of major human intestinal transport systems. The use of an *in vitro* model of human origin is particularly relevant to the aim of this manuscript, since rodents are poor surrogates for humans about OATPs expressed in the various tissues and the affinity and capacity of expressed OATPs for the transport of specific MC congeners (Feurstein et al., 2011).

The concentrations of MCs applied in the experiments were in the range, or even lower than the ones previously tested in other studies with cell lines in monolayer (Zeller et al., 2011; Vesterkvist et al., 2012; Henri et al., 2014) or with insect membrane vesicles overexpressing human transporters (Kaur et al., 2019). Based on the available data (Testai et al., 2016) on fish muscle (Cyprinidae) contamination**,** the 95th percentile of human fish consumption (based on the EFSA FoodEx database) would correspond to an ingestion of 94 μg MCs on average, with values up to the mg range, reached when highly contaminated fish or sea-food is assumed to be ingested. These values can be present in the lumen; for comparison, the absolute amount of MCs added to the AP compartment (mimicking the luminal side) was around 8 µg (when the highest concentration, i.e., 40 µM, was tested). Based on this data, the concentrations used here are fully representative of the actual exposure scenarios for humans.

The linearity vs. MC concentrations of the MC-RR, -LR and -YR detected in the BL compartment clearly indicated that, in the range of tested concentrations, none of the active transporters involved in their net absorption is saturated. In addition, it has been shown that the time of residence into the lumen is also an important variable, since MC-RR shows linearity vs. time with a rapid uptake and absorption, while the others are absorbed proportionally after a lag phase of 2 h. Looking at the Papp values, allowing to compare the rate of permeability of the 5 MCs, at 20 µM the more hydrophilic MCs, namely MC-RR, MC-YR and MC-LR, showed the highest Papp values vs. the more lipophilic ones (i.e., MC-LF and -LW). At variance, increasing the concentration up to 40 μM, all congeners have similar Papp values. When the % of absorption (assumed as the amount of parent compound detected in the BL compartment) is considered, in our experimental conditions, for MC-RR, -LR, -YR, the absorption was around 5% of the administered dose. For these three variants the toxins were present in trace or not detected in the pellet and the MB was >85%, suggesting that the possible binding to -SH groups in proteins, due to a nucleophilic reaction with other thiols within cells, is limited in our experimental conditions. The lipophilicity of -LW and -LF is likely responsible for the lower mass balance (>70% and <85%), also considering their detection within the cellular compartment (although at very low levels), likely associated to interaction with lipid membranes (Vesterkvist and Meriluoto, 2003). However, binding to proteins and adsorption to the plastic devices could not be excluded for these two variants.

Results of the kinetics of absorption using *ad hoc* cocktails of inhibitors indicate that oral absorption of MCs is a complex mechanism characterised by an equilibrium between absorption and extrusion, being MCs substrates of both influx and efflux proteins. Looking at the ER being significantly >1, the efflux system seems to be more relevant than the uptake in the gastrointestinal tract.

The intestinal absorption is only partially attributable to OATPs, since the use of the selected OATPs inhibitors has a limited impact on the uptake. This could be due to a combination of factors, amongst which the involvement of other influx proteins (e.g., OCT), or the overlapping inhibitor capacity of the used reference compounds resulting in enhanced opposite action on the efflux protein. As demonstrated with MC-LW and -LR, MCs are not P-gp substrates, whereas MRP2 is active in the extrusion, particularly for -LW, as representative of lipophilic variants.

Our results on the role of MRP2 are in line with Kaur et al. (2019), using insect membrane vesicles over-expressing the human transporters (BCRP, BSEP, MRP1,2,3,5, MDR1) to study MC transport. Kaur et al. (2019) found that the efflux by MRP2 was MC congener-specific, with MC-LF transported more rapidly than MC-LR and–RR applying MC concentrations ranging from 0.41μM to 1.8 mM. In the same study, other human transporters did not exhibit interaction with MCs. Conversely, in our system, a possible role for BCRP was shown, as evidenced by the inhibitory action of naringin on both OATP1A2 and BCRP, which failed to block the uptake and resulted in an increased net absorption.

The overlapping action of substrates and inhibitors on both influx and efflux transport systems is known and widely described in the literature (Kalliokoski and Niemi, 2009; Misaka et al., 2013; An et al., 2015; Yu et al., 2017). This represents the main hurdle to apply experimental models that are more complex than vesicles or specific transfected cellular models to study one transport system at a time. Moreover, An et al. (2015) hypothesised that, only when two chemicals share the same OATP binding site, the inhibition could be effective. In other words, a compound could be considered a specific OATP inhibitor vs*.* those substrates sharing the binding site, meaning that the selection of inhibitors can be substrate dependent and possibly clarified only by docking studies. This could be the case for MCs, since the selected OATP competitive inhibitors are not fully active.

The experimental system used in this manuscript is closer to the *in vivo* situation compared to most cell lines used *in vitro* to study intestinal absorption, particularly when the process involves a multi-transport system, as is here. Indeed, cell lines show an altered phenotypic feature with respect to the tissue of origin, implying a disequilibrium in the expression of several proteins. For example, Caco-2 cell line shares some characteristics with enterocytes, but do not originate from the small intestine and their representativeness of the intestinal epithelium is incomplete. However, data obtained with Caco-2 cells are qualitatively in line with our results. Henri et al. (2014) reported that in Caco-2 cell monolayers MC-LR (1, 10, 48 or 75 μM) was rapidly taken into cells from the AP (intestinal lumen) side. However, after 30–45 min, the majority of toxin was re-excreted back into the AP compartment, likely by efflux proteins, with only 0.3–1.35% of the toxin reaching the basolateral compartment over 24 h.

The biotransformation of MC has also been investigated through assessing the presence of MC-conjugates in the three compartments. Despite the presence of the GST protein, as an indication of metabolic competence of the intestinal experimental system, MC-conjugates were never detected. This may be due to 1) very low formation, undetectable by the HPLC system, 2) further metabolic steps to form MC-Cysteine conjugate, or 3) reversibility of the reaction leading to deconjugation to the parent toxin (Miles et al., 2016; Li et al., 2018). At present, no data on the transport of MC-conjugates are available, other than the finding that GSH-MC-LR conjugate added to insect membrane vesicles was not transported by MRP2, suggesting the involvement of transporters other than MRP2 for the conjugate efflux (Kaur et al., 2019).

Over the last 2 decades, efflux and influx transport proteins have become increasingly important due to their critical role in the kinetics of chemicals and this adds another level of complexity to the chemicals risk assessment process (Clerbaux et al., 2018). The complexity of the tissues used in this study with respect to other *in vitro* models provide a means to measure the net results of absorption (sum of influx and efflux passages) for compounds whose oral absorption feature depends on the relative affinity towards all the involved transporters. Such information can prove to be critical to investigate interaction between different compounds sharing affinities for a multiple-transporters. When *in vitro* systems are not fully representative of the *in vivo* transporter framework, results may lead to misinterpretation of chemical interactions and to biased predictions. In addition, once identified the role for individual transporters, the model used here could give, as a future step, the possibility to compare results from different donors: this may provide a means to investigate inter-individual differences in the transporters expression, including the presence of polymorphic variants and the relationships that may have with differences in individual susceptibility to toxicants. Although the contribution of transporter variability alone cannot be distinguished from other factors that can also contribute to variability in kinetic parameters, it has been recently reported that the 3.16 default kinetic Uncertainty Factor would cover healthy adult for inter-individual differences in transporters based on data for pharmaceutical probe substrates (Darney et al., 2020). However, data for the transport of environmental contaminants and food-relevant chemicals are very scarce, and therefore more research in this field is warranted for relevant compounds. The *in vitro* system presented here can contribute to investigating the role of transporters in xenobiotic absorption and extrusion. Such data can be integrated with other *in vitro* systems investigating isoform-specific phase I and phase II metabolism as well as *in silico* tools ultimately lead to more realistic QIVIVE and PBK models (Testai et al., 2021).

## Data Availability

The raw data supporting the conclusions of this article will be made available by the authors, without undue reservation.
