# Supplementary figures and images for: Congeners-Specific Intestinal Absorption Of Microcystins In An In Vitro 3D Human Intestinal Epithelium: The Role Of Influx/Efflux Transporters
Source: Front Toxicol. 2022 Aug 5;4:883063. doi: 10.3389/ftox.2022.883063 (PMC9388863; doi:10.3389/ftox.2022.883063)

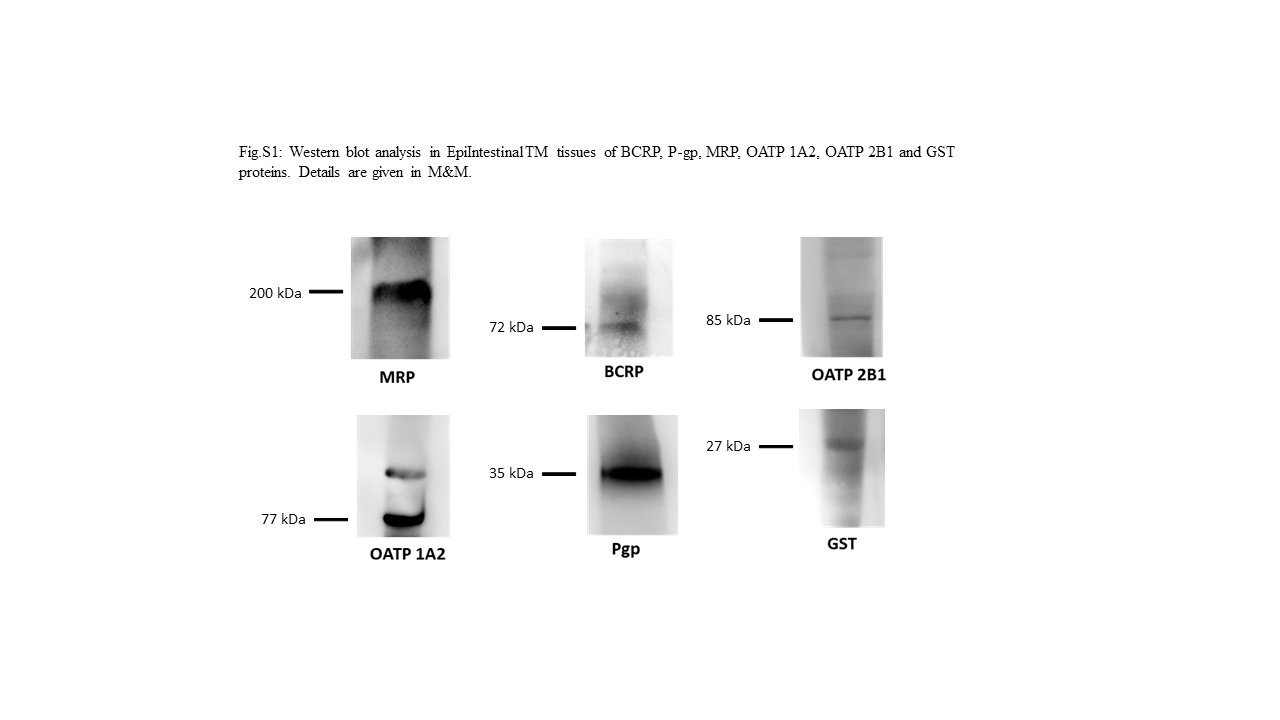

Supplement: Supplementary file 1 [file Image1.tif]
